# Supplementary material for: Liver Fibrosis Is Enhanced by a Higher Egg Burden in Younger Mice Infected with S. mansoni
Source: Cells. 2024 Oct 2;13(19):1643. doi: 10.3390/cells13191643 (PMC11475498; doi:10.3390/cells13191643)
Supplement: Supplementary file 1 [file cells-13-01643-s001.zip › cells-3213928-supplementary.pdf]

Supplementary material

Liver fibrosis is enhanced by a higher egg burden in younger mice infected with *S. mansoni*

Content:

Supplementary Figure S1: Infection outcome and mortality.
Supplementary Figure S2: Macroscopic pictures of livers from successfully-infected (right) and non-infected (left) mice.
Supplementary Figure S3: Scheme of the procedure for the assessment of relative granuloma area.
Supplementary Figure S4: Trending inverse correlation of liver-to-body weight ratio with ALT.
Supplementary Figure S5: Extramedullar hematopoiesis increased in the spleen of *S. mansoni*-infected older mice.
Supplementary Figure S6: Splenic granuloma around *S. mansoni* eggs.
Supplementary Figure S7: Hepatic inflammatory infiltration in direct vicinity of *S. mansoni* worms.
Supplementary Figure S8: *S. mansoni* infection-induced pronounced expression of *Il-12* in young mice.
Supplementary Figure S9: Hepatic *Il-10* expression levels moderately correlated to hepatic eggload.
Supplementary Figure S10: Hepatic *Il-23* mRNA expression.
Supplementary Figure S11: Enhanced hepatic type III collagen expression in *S. mansoni* infected mice.
Supplementary Figure S12: Enhanced histologic staining of fibrillar collagens in *S. mansoni*-infected mice.
Supplementary Figure S13: *S. mansoni* infection-induced pronounced expression of *Pdgf B* and *Tgf-β* in younger mice.
Supplementary Figure S14: Desmin immunostaining of liver slices from *S. mansoni* infected mice.
Supplementary Figure S15: Validation of the appearance of desmin$^+$ cells in hepatic granuloma of a patient infected with *S. mansoni*.
Supplementary Figure S16: *S. mansoni* eggs directly stimulate *Hk2* and *Pkm2* in primary mouse hepatocytes without an immune reaction against the parasite.
Supplementary Figure S17: *S. mansoni* infection reduced the hepatic expression of *Plin2* and *Fasn*.
Supplementary Figure S18: *S. mansoni* infection caused a drop of hepatic *Cat* levels.
Supplementary Table S1: Primer sequences.

Suppl. Fig. 1

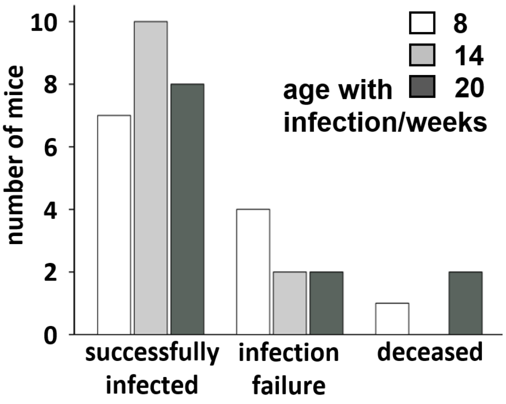

Supplementary Figure S1: Infection outcome and mortality. Bars indicate the numbers of successful infections and mortality. Colour code indicates the age of the mice at the time of infection: white 8 weeks, light grey 14 weeks, dark grey 20 weeks. Chi-square test: X^2=0.47.

Suppl. Fig. 2

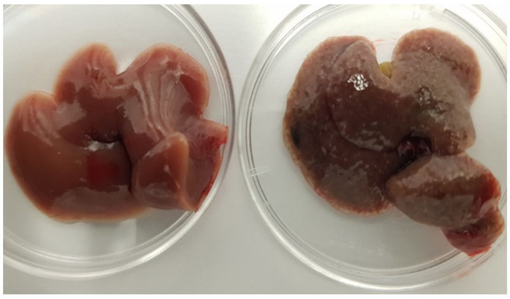

Supplementary Figure 2: Macroscopic pictures of livers from successfully-infected (right) and non-infected (left) mice.

Suppl. Fig. 3

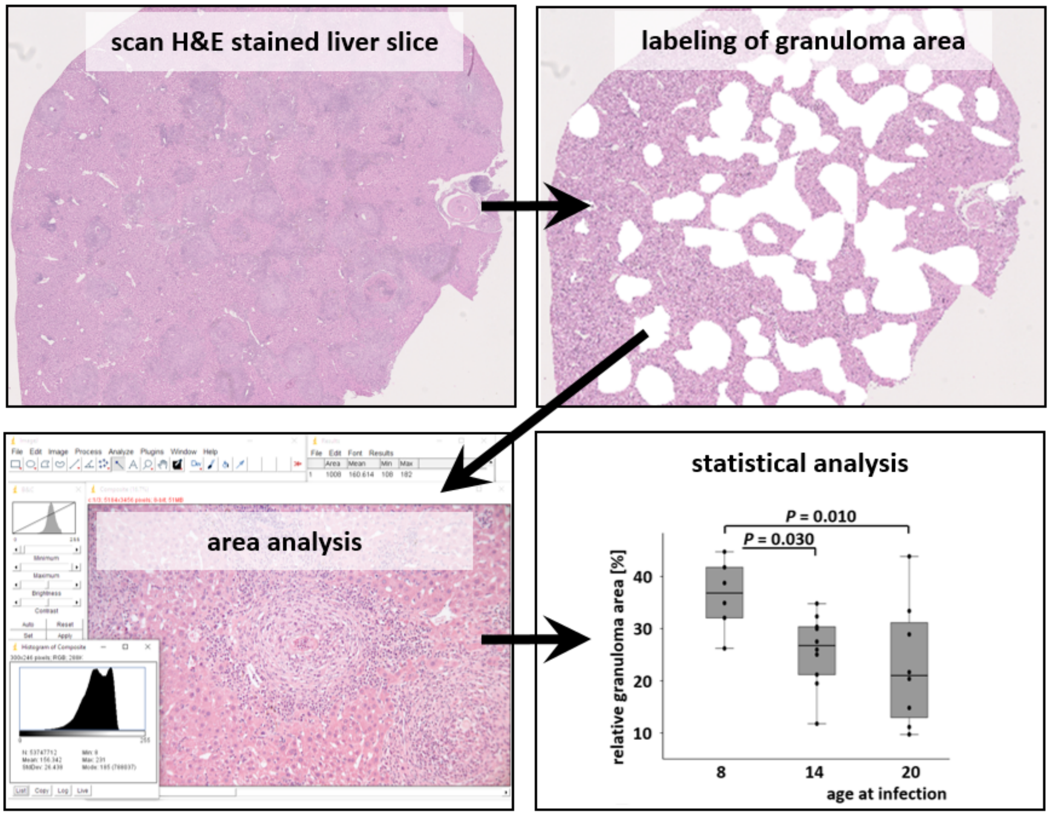

Supplementary Figure 3: Scheme of the procedure for the assessment of relative granuloma area. A high resolution scan of complete liver slices from each mouse was assessed (upper left). Granuloma area were colour labelled (upper right) and quantified in ImageJ, version 1.51 (lower left). Relative granuloma areas of a representative cross-section may be a measure for relative granuloma extent in the whole liver. One-way ANOVA and post hoc Fisher's LSD was used for pairwise comparison of groups. Statistics and design of the bar graph was done in SPSS29 (lower right corresponds to Fig. 1 B).

Suppl. Fig. 4

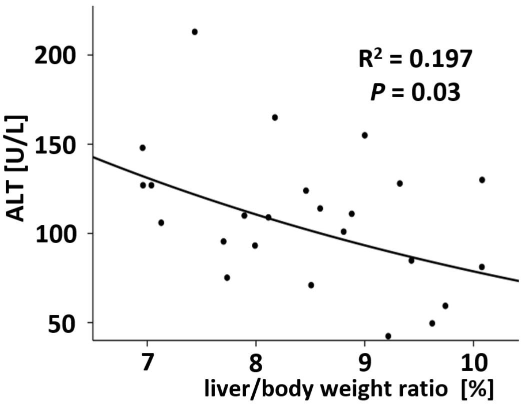

Supplementary Figure 4: Trending inverse correlation of liver-to-body weight ratio with ALT.

Suppl. Fig. 5

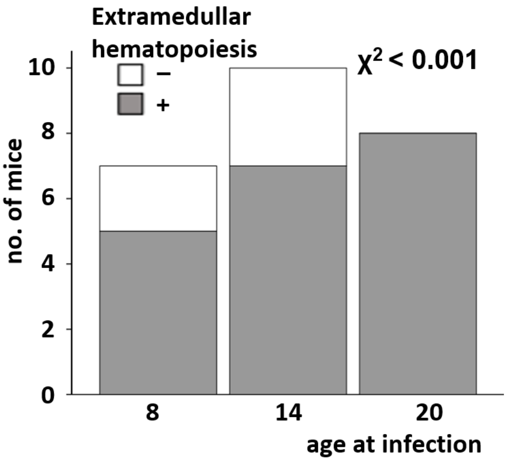

Supplementary Figure S5: Extramedullar hematopoiesis increased in the spleen of *S. mansoni*-infected older mice.

Suppl. Fig. 6

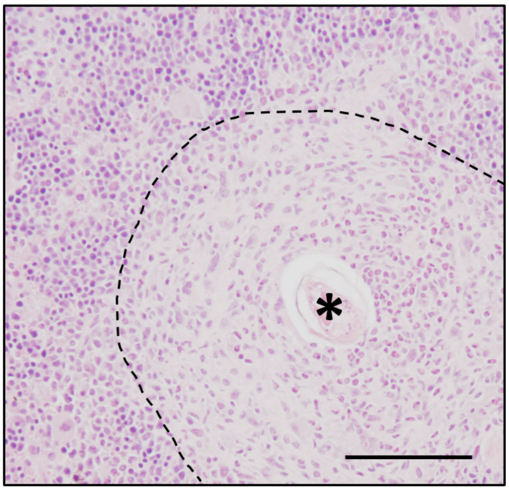

Supplementary Figure S6: Splenic granuloma around *S. mansoni* eggs. Representative H&E stained liver slice, 200x, bar 100 μm.

Suppl. Fig. 7

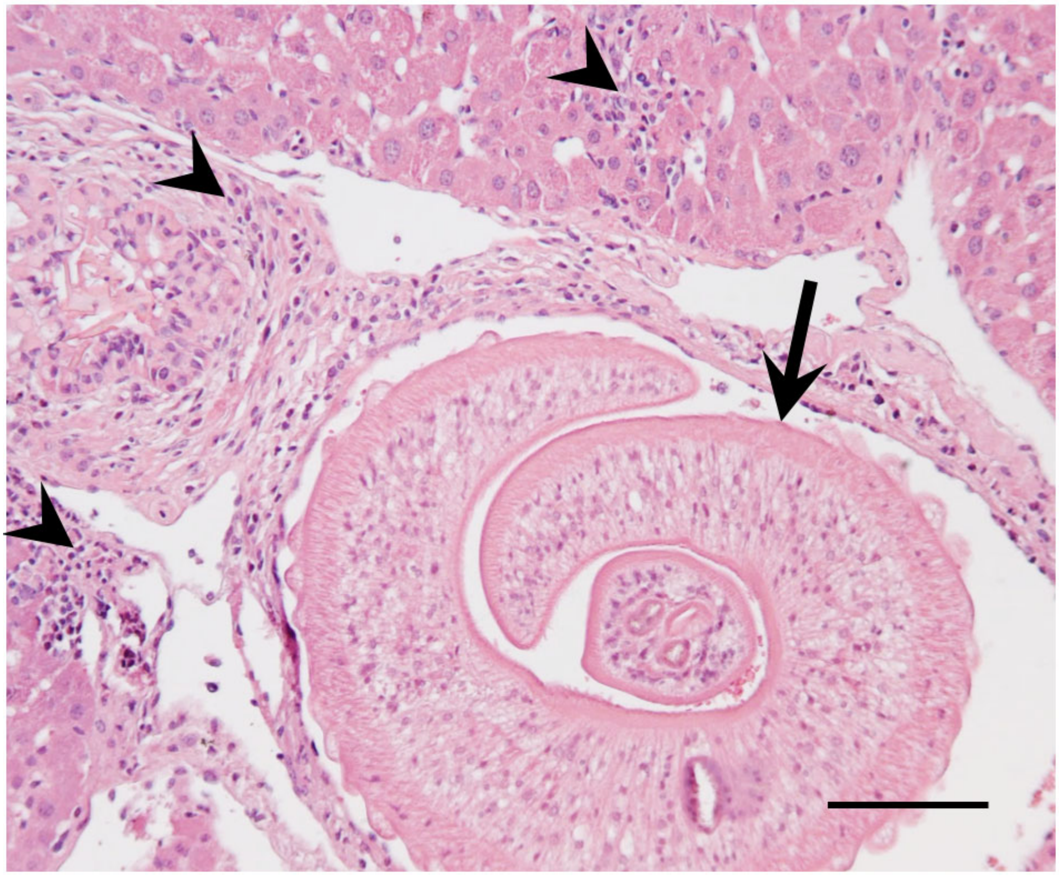

Supplementary Figure S7: Hepatic inflammatory infiltration in direct vicinity of *S. mansoni* worms. Arrow indicates an *S. mansoni* worm in a large branch of the portal vein. Arrowheads indicate inflammatory infiltration in the surrounding host tissue. Representative H&E stained liver slice, bar 100 µm.

Suppl. Fig. 8

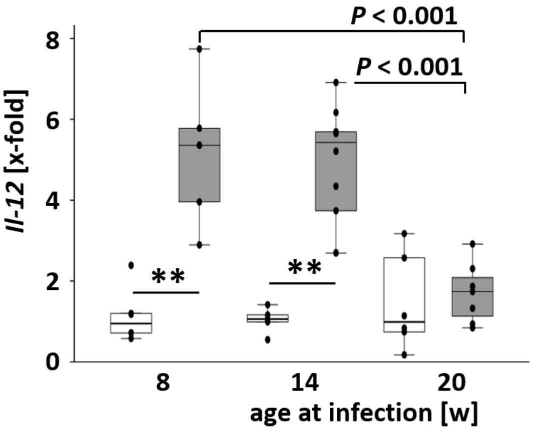

Supplementary Figure S8: ***S. mansoni*** **infection-induced pronounced expression of** ***Il-12*** **in young mice.** *S. mansoni*-induced *Il-12* expression is lower in 20 weeks old mice. **P<0.001, white bars uninfected mice, grey bars infected mice. The indicated P values were calculated by ANOVA and post hoc pairwise comparison of groups using Fisher's LSD.

Suppl. Fig. 9

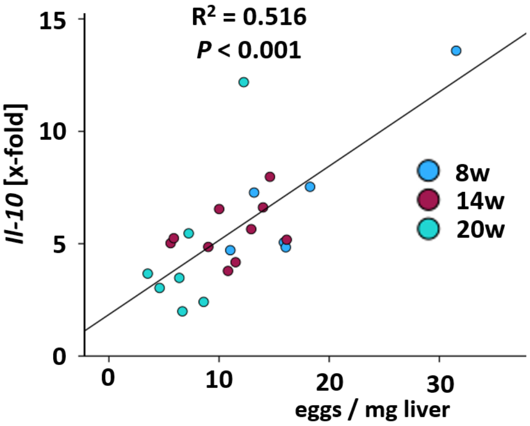

Supplementary Figure S9: Hepatic *Il-10* expression levels moderately correlated to hepatic eggload.

Suppl. Fig. 10

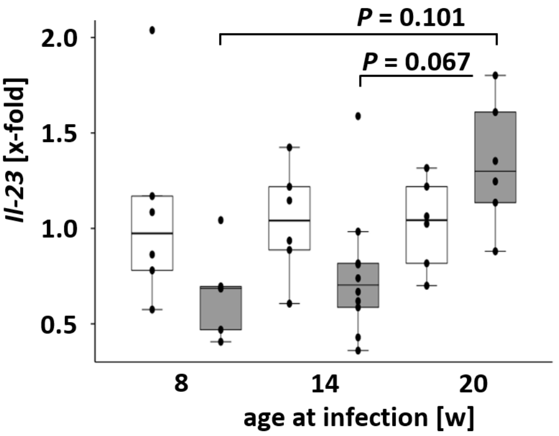

Supplementary Figure S10: Hepatic *Il-23* mRNA expression. White bars uninfected mice, grey bars infected mice. The indicated P values were calculated by ANOVA and post hoc pairwise comparison of groups using Fisher's LSD.

Suppl. Fig. 11

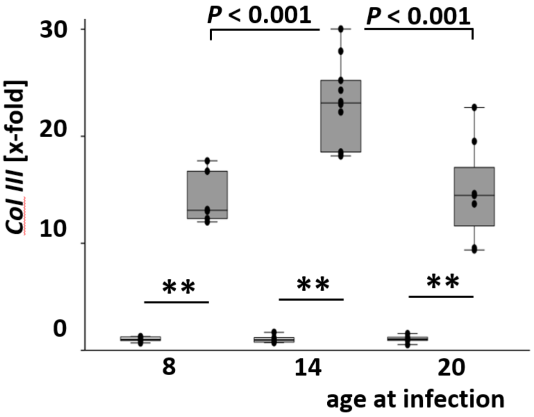

Supplementary Figure S11: Enhanced hepatic type III collagen expression in *S. mansoni* infected mice. Type III collagen was induced on transcriptional level by *S. mansoni* infection with a maximum in mice infected at the age of 14 weeks. **P<0.001, white bars uninfected mice, grey bars infected mice. The indicated P values were calculated by ANOVA and post hoc pairwise comparison of groups using Fisher's LSD.

Suppl. Fig. 12

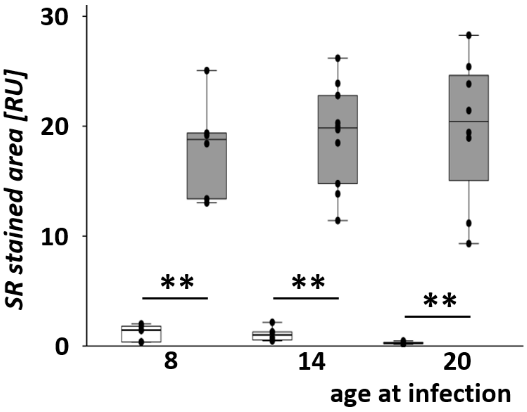

Supplementary Figure S12: Enhanced histologic staining of fibrillar collagens in *S. mansoni*-infected mice. Sirius red stained area was enhanced in *S. mansoni*-infected mice. Morphometric analysis revealed an equal increase of Sirius red stained area in all three groups of infected mice, regardless of the age of infection. **P<0.001, white bars uninfected mice, grey bars infected mice. The indicated P values were calculated by ANOVA and post hoc pairwise comparison of groups using Fisher's LSD.

Suppl. Fig. 13

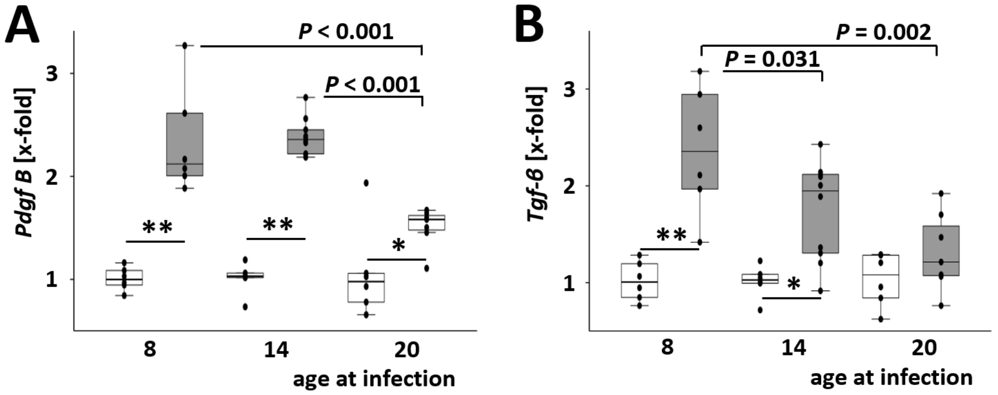

Supplementary Figure S13: *S. mansoni* infection-induced pronounced expression of *Pdgf B* and *Tgf-β* in younger mice. Hepatic *Pdgf B* (A) and *Tgf-β* (B) were induced by *S. mansoni* infection but dropped with increasing host age in infected animals. *P<0.05, **P<0.001, white bars uninfected mice, grey bars infected mice. The indicated P values were calculated by ANOVA and post hoc pairwise comparison of groups using Fisher's LSD.

Suppl. Fig. 14

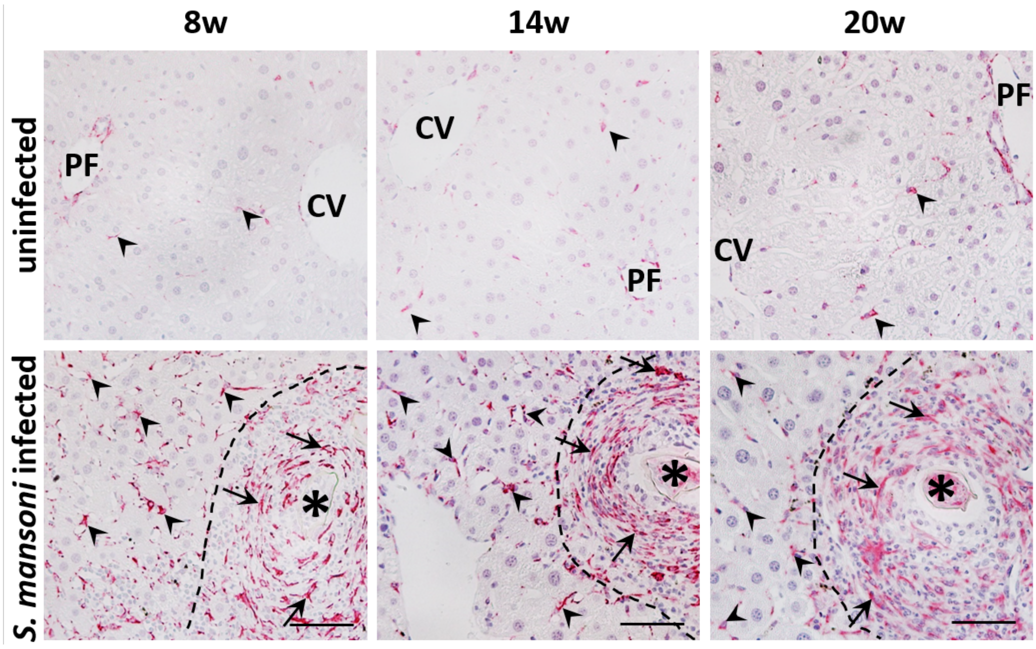

Supplementary Figure S14: Desmin immunostaining of liver slices from *S. mansoni*-infected mice. The number of Desmin-positive cells was raised in the hepatic parenchyma of *S. mansoni*-infected mice in comparison to uninfected controls (arrowheads). Desmin positive cells appeared in high numbers and density inside the granulomas (arrows). *eggs, dashed line – border of granuloma. Representative immunostainings are shown, magnification 200x, bar 100µm.

Suppl. Fig. 15

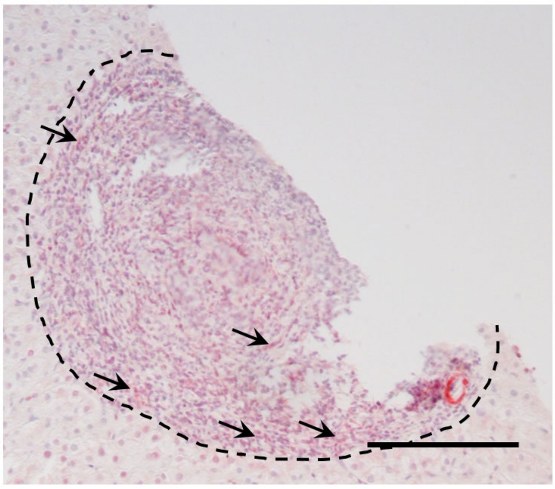

Supplementary Figure S15: Validation of the appearance of desmin+ cells in hepatic granuloma of a patient infected with *S. mansoni*. Histologic specimen of a colon biopsy from a 31 year old male patient with schistosomiasis was stained for desmin. Desmin positive cells appeared in high numbers and density inside the granulomas (arrows). Dashed line – border of granuloma. Representative immunostaining is shown, magnification 200x, bar 100μm.

Suppl. Fig. 16

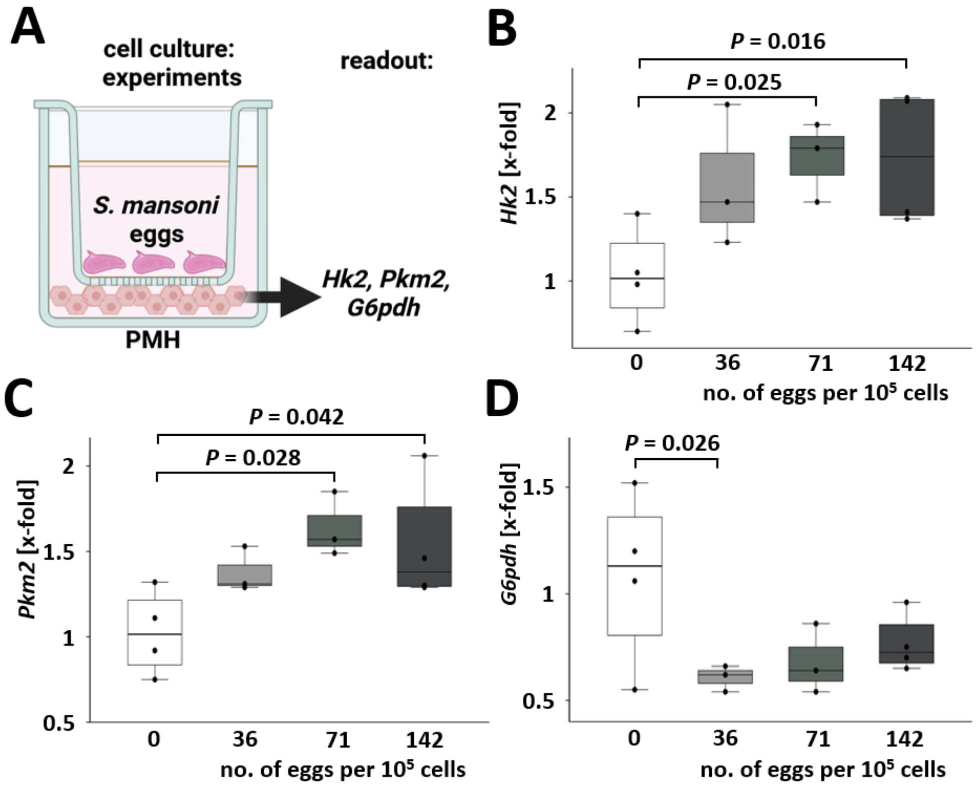

Supplementary Figure S16: ***S. mansoni*** **eggs directly stimulate the expression of** ***Hk2*** **and** ***Pkm2*** **in primary mouse hepatocytes without an immune reaction against the parasite.** (A) Primary mouse hepatocytes (PMH) were cocultivated with increasing numbers of *S. mansoni* eggs in a 6-well transwell chamber system. *Hk2* (B) and *Pkm2* (C) increased, while *G6pdh* (D) was reduced in *S. mansoni* egg-stimulated PMH. 3.5×10^5 cells per well, Transwell: Falcon #3493. **P<0.001, white bars uninfected mice, grey bars infected mice. The indicated P values were calculated by ANOVA and post hoc pairwise comparison of groups using Fisher's LSD.

Suppl. Fig. 17

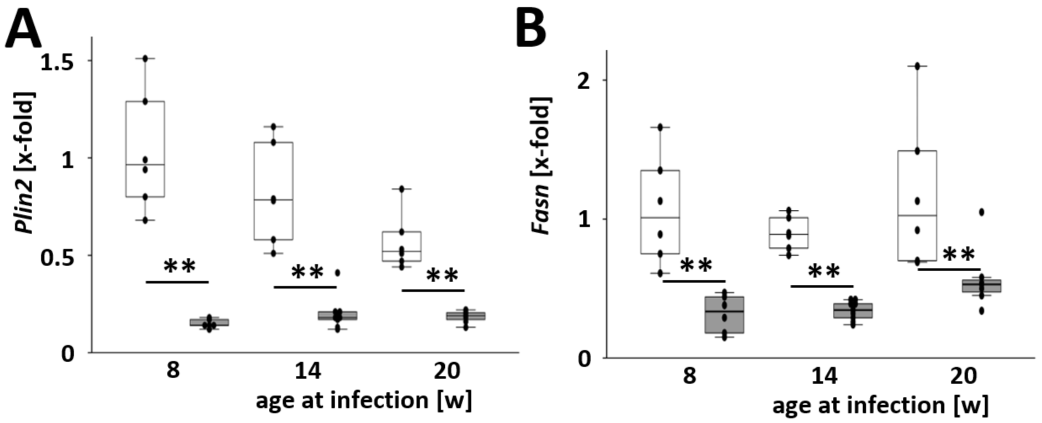

Supplementary Figure S17: ***S. mansoni*** **infection reduced the hepatic expression of** ***Plin2*** **and** ***Fasn*.** **P<0.001, white bars uninfected mice, grey bars infected mice. The indicated P values were calculated by ANOVA and post hoc pairwise comparison of groups using Fisher's LSD.

Suppl. Fig. 18

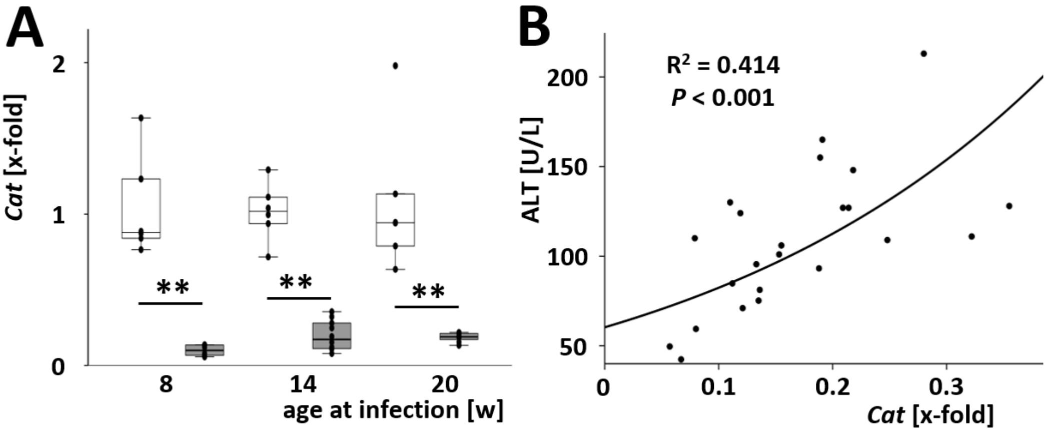

Supplementary Figure S18: *S. mansoni* infection caused a drop of hepatic *Cat* levels. (A) *Cat* expression was downregulated in the liver of *S. mansoni* infected mice. (B) Hepatic *Cat* levels were positively correlated with serum ALT in *S. mansoni*-infected mice. *Cat* catalase mRNA. **P<0.001, white bars uninfected mice, grey bars infected mice. The indicated P values were calculated by ANOVA and post hoc pairwise comparison of groups using Fisher's LSD (A) or logistic regression analysis (B).

Suppl. Tab. 1: Primers used were:

Il-4 (sense: 5'-ggt ctc aac ccc cag cta gt-3', antisense: 5'-gcc gat gat ctc tct caa gtg at-3'), Il-6 (sense: 5'-tcc agt tgc ctt ctt ggg ac-3', antisense: 5'-gta ctc cag aag acc aga gg-3'), Il-10 (sense: 5'-ccc att cct cgt cac gat ctc-3', antisense: 5'-tca gac tgg ttt ggg ata ggt tt-3'), , Tnf-α (sense: 5′ –gcc cac gtc gta gca aac cac- 3′, antisense: 5′ –gca ggg gct ctt gac ggc ag- 3′), Inf-γ (sense: 5′ -ctg cat ctt ggc ttt gca gc- 3′, antisense: 5′ -aga taa tct ggc tct gca gga t- 3′), Il-12 (sense: 5′ -cat cag gga cat cat caa acc a- 3′, antisense: 5′ -aag gtg cgt tcc tcg tag ag- 3′), Il-23 (sense: 5′ -- 3′, antisense: 5′ -- 3′), Col 1 (sense: 5′ -gct cct ctt agg ggc cac t-3′, antisense: 5′ -cca cgt ctc acc att ggg g- 3′), Col 3 (sense: 5′ – gcc cac agc ctt cta cac- 3′, antisense 5′ -cca ggg tca cca ttt ctc- 3′), Cd45 (sense: 5′ -gat gtc agt tgg aca acc ttc g- 3′, antisense: 5′-gat cag gtt tag atg cag gct c-3′), Hk2 (sense: 5' -aag gtg gaa atg gag cag gg-3', antisense: 5' -ccc gga agt ttg ttc ctc ca-3'), Pkm2 (sense: 5' -tca ccc tgg aca acg ctt ac- 3', antisense: 5' -agt cag cgc ctt tct cct tc- 3'), G6pdh (sense: 5′ -tcc tac cat ctg gtg gct gt- 3′, antisense: 5′ -gca aag aac tcc tcc agc tt- 3′).
